# Supplementary material for: ClinGen guidance for use of the PP1/BS4 co-segregation and PP4 phenotype specificity criteria for sequence variant pathogenicity classification
Source: Am J Hum Genet. 2023 Dec 15;111(1):24–38. doi: 10.1016/j.ajhg.2023.11.009 (PMC10806742; doi:10.1016/j.ajhg.2023.11.009)
Supplement: Table S1. Worksheet for apportioning evidence among two plausible candidate variants on an allele that is implicated by PP1 or PP4 evidence [file mmc1.pdf]

The American Journal of Human Genetics, Volume *111*

Supplemental information

ClinGen guidance for use of the PP1/BS4 co-segregation and PP4 phenotype specificity criteria for sequence variant pathogenicity classification

Leslie G. Biesecker, Alicia B. Byrne, Steven M. Harrison, Tina Pesaran, Alejandro A. Schäffer, Brian H. Shirts, Sean V. Tavtigian, Heidi L. Rehm, and ClinGen Sequence Variant Interpretation Working Group

Table S1. Worksheet for apportioning evidence amongst two plausible candidate variants on an allele that is implicated by PP1 or PP4 evidence.

Var1 evidence criteria: ___________________________ (list criteria, e.g., PVS1_Moderate, but do NOT include PP1, PP4, or BS4 evidence

Var1 evidence points total: _________pts Line 1

Var1 posterior probability % (Table 2) based on points from Line 1 _________% Line 2

Var2 evidence criteria: ___________________________ (list criteria, e.g., PP3_Mod, but do NOT include PP1, PP4, or BS4 evidence

Var 2 evidence points total: _________pts Line 3

Var2 posterior probability % (Table 2) based on points from Line 1 _________% Line 4

Divide (Line 2)/(Line 4), (relative pathogenicity of Var1 to Var2) _________:1 Line 5

Enter the diagnostic yield of testing for this phenotype: _________% Line 6

Adjusted posterior for Var1 = (Line 6) * (Line 5)/((Line 5) +1) _________% Line 7

Convert Var1 adjusted posterior to points, using Table 2 _________pts Line 8

Adjusted posterior for Var2 = (Line 6) * (1/(Line 5)/(1/Line 5 +1) _________% Line 9

Convert Var2 adjusted posterior to points, using Table 2 _________pts Line 10

Error check: Add (Line 7) + (Line 9), this should be equal to Line 6 _________%
