# Supplementary material for: ClinGen guidance for use of the PP1/BS4 co-segregation and PP4 phenotype specificity criteria for sequence variant pathogenicity classification
Source: Am J Hum Genet. 2023 Dec 15;111(1):24–38. doi: 10.1016/j.ajhg.2023.11.009 (PMC10806742; doi:10.1016/j.ajhg.2023.11.009)
Supplement: Document S1. Article plus supplemental information [file mmc2.pdf]

ClinGen guidance for use of the PP1/BS4 co-segregation and PP4 phenotype specificity criteria for sequence variant pathogenicity classification

Authors

Leslie G. Biesecker, Alicia B. Byrne, Steven M. Harrison, ..., Sean V. Tavtigian, Heidi L. Rehm, ClinGen Sequence Variant Interpretation Working Group

Correspondence

lesb@mail.nih.gov

The 2015 ACMG/AMP variant classification recommendations included pathogenic (PP1) and benign (BS4) criteria for co-segregation, but details for using them were sparse. We developed a points-based co-segregation heuristic and integrated the specific phenotype criterion (PP4). We also show that negative evidence at one locus constitutes positive evidence for other loci.

Check for updates

Biesecker et al., 2024, The American Journal of Human Genetics *111*, 24–38
January 4, 2024 © 2023
https://doi.org/10.1016/j.ajhg.2023.11.009

CellPress

ARTICLE

ClinGen guidance for use of the PP1/BS4 co-segregation and PP4 phenotype specificity criteria for sequence variant pathogenicity classification

Leslie G. Biesecker,[1,*] Alicia B. Byrne,[2] Steven M. Harrison,[2,3] Tina Pesaran,[3] Alejandro A. Schäffer,[4] Brian H. Shirts,[5] Sean V. Tavtigian,[6] Heidi L. Rehm,[2,7] and ClinGen Sequence Variant Interpretation Working Group

Summary

The 2015 American College of Medical Genetics and Genomics and the Association for Molecular Pathology variant classification publication established a standard employed internationally to guide laboratories in variant assessment. Those recommendations included both pathogenic (PP1) and benign (BS4) criteria for evaluating the inheritance patterns of variants, but details of how to apply those criteria at appropriate evidence levels were sparse. Several publications have since attempted to provide additional guidance, but anecdotally, this issue is still challenging. Additionally, it is not clear that those prior efforts fully distinguished disease-gene identification considerations from variant pathogenicity considerations nor did they address autosomal-recessive and X-linked inheritance. Here, we have taken a mixed inductive and deductive approach to this problem using real diseases as examples. We have developed a practical heuristic for genetic co-segregation evidence and have also determined that the specific phenotype criterion (PP4) is inseparably coupled to the co-segregation criterion. We have also determined that negative evidence at one locus constitutes positive evidence for other loci for disorders with locus heterogeneity. Finally, we provide a points-based system for evaluating phenotype and co-segregation as evidence types to support or refute a locus and show how that can be integrated into the Bayesian framework now used for variant classification and consistent with the 2015 guidelines.

Introduction

The American College of Medical Genetics and Genomics and the Association for Molecular Pathology (ACMG/AMP) established a set of consensus guidelines for evaluating evidence for the pathogenicity of genomic variants.[1] In those guidelines, co-segregation of a variant with the phenotype was considered supporting evidence for pathogenicity (designated by the alphanumeric criterion code PP1), and observation of non-segregation of a variant with disease was considered strong evidence against pathogenicity (benign criterion BS4). ACMG/AMP also noted that PP1 "may be used as stronger evidence with increasing segregation data," although the methods to do that were not specified. We set out to evaluate genetic co-segregation as a pathogenicity criterion with a Bayesian framework.[2] Our goal was to develop practical recommendations for the PP1, BS4, and PP4 criteria that address commonly encountered clinical testing scenarios. Here, we developed a pragmatic proposal (Figure 1) for integrating and weighting co-segregation evidence (PP1 and BS4) and phenotype evidence (PP4) in a two-tiered approach that (1) provides points-based application of simple co-segregation data and (2) refers to formal, maximal likelihood ratio calculations when the data are extensive or complex.

Material and methods

This working group was convened as an informal subcommittee of the Clinical Genome Resource (ClinGen)[3] Sequence Variant Interpretation Working Group (https://www.clinicalgenome.org/working-groups/sequence-variant-interpretation/). This group undertook a series of monthly conference calls and a few in-person meetings beginning in August 2018. In this work, we used both inductive and deductive reasoning derived from principles that are long established in human genetics plus carefully chosen examples of real genetic disorders to illustrate and inform our reasoning. The variants that we use in these examples (details presented in Table 1) are either observed in gnomAD or hypothetical. We specifically focused on organizing and evaluating evidence based on the Bayesian adaptation[2] and subsequent points-based adaptation[4] of Richards et al.[1] Briefly, this method uses a naive Bayesian classifier to calculate the effect of evidence for or against pathogenicity based on the criteria of Richards et al. A prior probability of pathogenicity (0.102) is assigned to the variant, and the pathogenicity or benignity evidence criteria are treated as conditional probabilities, expressed as odds of pathogenicity ($\text{Odds}_{\text{path}}$), which are then used to calculate the posterior probability of pathogenicity.

The target audience for these recommendations includes clinical genetic and genomic testing laboratory directors and staff as well as rare disease researchers who are classifying variant

[1]Center for Precision Health Research, National Human Genome Research Institute, National Institutes of Health, Bethesda, MD, USA; [2]Program in Medical and Population Genetics, Broad Institute of MIT and Harvard, Cambridge, MA, USA; [3]Ambry Genetics, Aliso Viejo, CA, USA; [4]Cancer Data Science Laboratory, National Cancer Institute, National Institutes of Health, Bethesda, MD, USA; [5]Department of Laboratory Medicine and Pathology, University of Washington, Seattle, WA, USA; [6]Department of Oncological Sciences, University of Utah School of Medicine and Huntsman Cancer Institute at the University of Utah, Salt Lake City, UT, USA; [7]Center for Genomic Medicine, Massachusetts General Hospital, Boston, MA, USA
*Correspondence: lesb@mail.nih.gov
https://doi.org/10.1016/j.ajhg.2023.11.009.
© 2023 This is an open access article under the CC BY license (http://creativecommons.org/licenses/by/4.0/).

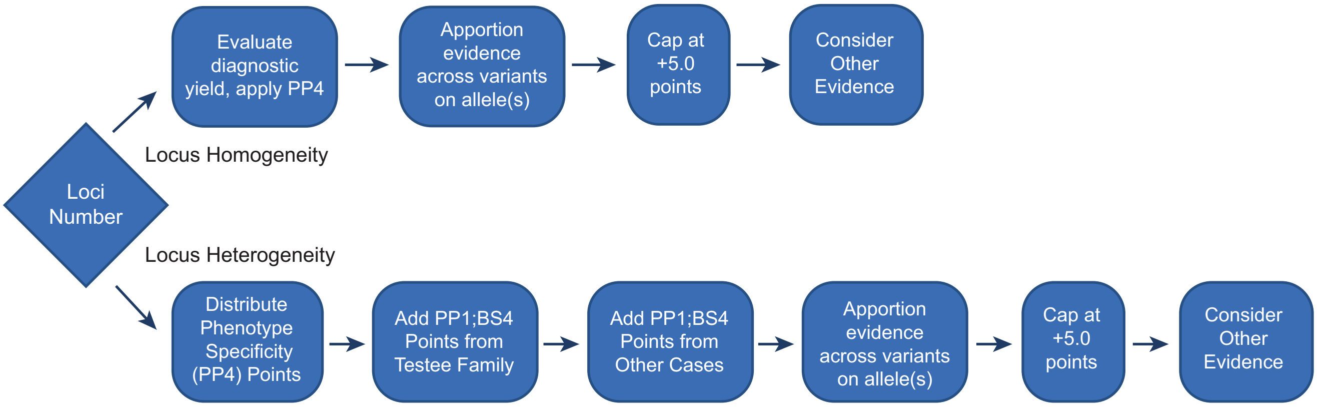

Figure 1. Flow diagram showing steps of evidence assessment for the phenotype-specificity (PP4) and co-segregation (PP1/BS4) criteria

pathogenicity in genes associated with Mendelian disorders that have been assessed as having definitive or strong gene-disease validity.[6] The recommendations should be used with caution in any gene with a lower gene-disease validity or low penetrance (e.g., susceptibility variants) and should not be used for the determination of novel gene-disease associations. In applying these recommendations, evidence may be incorporated from the family currently being tested and previously reported families with data that can be used to assess co-segregation evidence for the same variant. It is important that the individuals using these recommendations have a sound understanding of the principles of Mendelian inheritance so that they will be able to properly apply these recommendations and recognize their limitations.

We use the terms co-segregation and segregation analysis in this manuscript, which have been used in prior publications including Richards et al.[1] We acknowledge that the term segregation analysis is also used to describe the assessment of inheritance patterns in multiplex families, which is a distinct analysis. Linkage terminology, consistent with that used in current textbooks and seminal publications on linkage analysis, could be used to describe more precisely some scenarios below; indeed, formal co-segregation analysis for variant classification can be statistically modeled as a specific case of linkage analysis.[7] However, we will use the terms co-segregation and segregation analysis when referring to evaluation of variant and disease occurring together to be consistent with most variant classification literature. We use the term "linkage" to refer to formal linkage analysis and analysis of genes or variants occurring together.[8]

Results

We started with a simple example, which may not be representative of a substantial number of clinical diagnostic cases but nonetheless illustrates important principles of co-segregation evidence (PP1 and BS4) and the conceptual relationship of these to the phenotype criterion (PP4). We then modeled a series of scenarios, changing as few variables as possible to learn how co-segregation affected our assessment of pathogenicity, using a combination of inductive and deductive approaches to derive general principles and to test examples.

Locus homogeneity and its relationship to co-segregation evidence

We chose the disease in the initial example to be *CTNS*-related cystinosis (MIM: 219750), which has autosomal-recessive inheritance due to biallelic pathogenic variants in *CTNS* as well as locus homogeneity.[9,10] There are no patients with the typical cystinosis phenotype known to have the condition due to variants in any other gene (W. Gahl, personal communication). The diagnostic yield of pathogenic variants in this gene for individuals with a clinical diagnosis of typical cystinosis is 95.8%.[11] This analysis led us to several conclusions.

The key conclusion is that it is not possible to generate co-segregation evidence in support of the pathogenicity of a *CTNS* variant because all variants in this gene must be nearly perfectly linked to the phenotype by virtue of the high specificity of that phenotype coupled with the locus homogeneity. Any two variants in *trans* identified in an affected individual have a prior probability of essentially 100% to co-segregate with the phenotype in an affected family. If the prior probability of the gene's role in the affected individual's phenotype is nearly 100%, the observation of co-segregations of the phenotype and the observed genetic variants cannot provide additional (significant) conditional odds of pathogenicity (Figure 2A).

This counterintuitive example led us to recognize that the phenotype criterion PP4 is conceptually and inextricably coupled to the co-segregation criterion PP1, which would violate the requirements of a naive Bayesian classifier. In the *CTNS*-related cystinosis example of autosomal-recessive inheritance for a disorder with locus homogeneity, high penetrance, and a near-zero phenocopy rate, the specification of this phenotype is essentially equivalent to an infinitely high LOD score—co-segregation will always be perfect and is theoretically infinite. We concluded that if a trait exhibits locus homogeneity and a high diagnostic yield of testing where the evidence for the diagnostic yield is methodologically comparable to the clinical testing methodology, PP4 implicates the pathogenicity of allele(s) at that locus with a high conditional

Table 1. **Details of the variants used in examples in this publication**

Gene	MIM	MANE transcript	GRCh38	cDNA change	Protein change	REVEL	gnomAD Count
CTNS	606272	GenBank: NM_004937.3	Chr17:3,640,208T>G	c.2T>G	p.Met1Arg	0.650	0
CTNS	–	–	Chr17:3,647,477T>G	c.95T>G	p.Val32Gly	0.698	0
CTNS	–	–	Chr17:3,647,492G>C	c.110G>C	p.Gly37Ala	0.211	0
ERCC6	609413	GenBank: NM_000124.4	Chr10:49,470,196A>G	c.3764T>C	p.Leu1255Pro	0.939	0
ERCC6	–	–	Chr10:49,493,174C>A	c.1764G>T	p.Trp588Cys	0.951	1
ERCC8	609412	GenBank: NM_000082.4	Chr5:60,918,365T>A	c.287A>T	p.Tyr100Phe	0.070	0
FBN1	134797	GenBank: NM_000138.5	Chr15:48,489,919T>A	c.3014A>T	p.Glu1005Val	0.432	1
FBN1	–	–	Chr15:48,492,496C>A	c.2819T>G	p.Gly940Val	0.931	1
TSC1	605284	GenBank: NM_000368.5	Chr9:132,928,817A>T	c.56T>A	p.Leu19Gln	0.791	1
TSC2	191092	GenBank: NM_001114382	Chr16:2,048,716A>C	c.101A>C	p.Lys34Thr	0.153	2
MYH7	160760	GenBank: NM_000257.4	Chr14:23,417,174G>A	c.4498C>T	p.Arg1500Trp	0.836	0
RBM10	300080	GenBank: NM_005676.5	ChrX:47,176,589G>T	c.656G>T	p.Cys219Phe	0.928	0

REVEL scores are from Ioannidis et al.[5]

$Odds_{path}$. The implication of this deduction is that disorders with locus homogeneity and high diagnostic yield can generate robust evidence of pathogenicity (as per PP4 but with a higher level of strength than supporting), and it would be inappropriate to apply co-segregation evidence (PP1). An example of this scenario is shown in Figure 2A.

Capping locus evidence when it is used for variant pathogenicity evidence

We affirmed the qualitative recognition by Richards et al.[1] (and others) of a limit to this form of evidence because of the possibility that an observed variant in a given family may not actually be pathogenic but instead be in linkage disequilibrium with the pathogenic variant. A well-known example of this is the *CFTR* c.443T>C (p.Ile148Thr) variant (MIM: 602421), which was presumed to be pathogenic and later found to be benign but in linkage disequilibrium (LD) with the actual causative variant c.3067_3072delATAGTG (p.Ile1023_Val1024del).[12] We recognized that even though PP1 and PP4 could theoretically yield a high conditional $Odds_{path}$, there is always a possibility of another variant in LD and that a robust classification of likely pathogenic or pathogenic should be based on additional evidence outside of PP1 and PP4, such as PVS1 (predicted loss of function[13]), PS3 (functional data[14]), or PP3 (*in silico*[15]), i.e., evidence that implicates a specific variant. Therefore, we recommend that pathogenicity evidence that formally implicates a locus (PP4 and PP1) should be capped at +5.0 points (conditional $Odds_{path}$ of 38.9:1), where +6.0 points (conditional $Odds_{path}$ of 81.0:1) is necessary to reach a posterior probability of likely pathogenic (90%) and +10.0 points (conditional $odds_{path}$ of 1,516:1) is necessary to reach pathogenic (99%).[4] An exception would be a disorder where pathogenic variation at the locus must be coding (e.g., gain-of-function mechanism) and all coding regions have been sequenced, which is discussed below. For the hypothetical example of the *CTNS* p.Met1Arg and p.Val55Gly variants identified in an affected individual (Figure 2A), we reasoned that the phenotype specificity of the disorder leads to a high posterior probability of pathogenicity at the gene level, which in autosomal-recessive inheritance implicates both variants. To facilitate the assessment of the strength of this evidence, we have created a table of values that allows analysts to convert mutational yield (a hypothetical posterior) to a Bayesian points value that can be assigned to the allele/variant (Table 2). Given the 95.8% diagnostic yield for this gene for this phenotype, it would fall between the 95% and 96.5% values on Table 2, which corresponds to +7.0 and +7.5 points, respectively (and we round down to the lower point value if the yield falls between two values). Per the cap proposed above, both the p.Met1Arg and p.Val55Gly variants earn +5.0 points. The two alleles in this compound heterozygous case are both awarded the full +5.0 points because this evidence is not divided or apportioned between the two alleles. This is because this is gene-based evidence and therefore the full evidence applies to both alleles. Were the case to have been homozygous, the evidence for the (single) variant would be the same +5.0 points, again because it is gene-based evidence.

Locus and allele evidence and how they relate to variant evidence

Variants in a gene for a disorder with locus homogeneity, high penetrance, low phenocopy rate, and high diagnostic yield (like *CTNS*) will show evidence of perfect segregation with the trait. Benign missense variants, synonymous variants, regulatory variants, and intronic variants will similarly be linked to the trait. One implication of this is that the evidence in support of the pathogenicity of the allele must be distributed among the relevant, observed variants

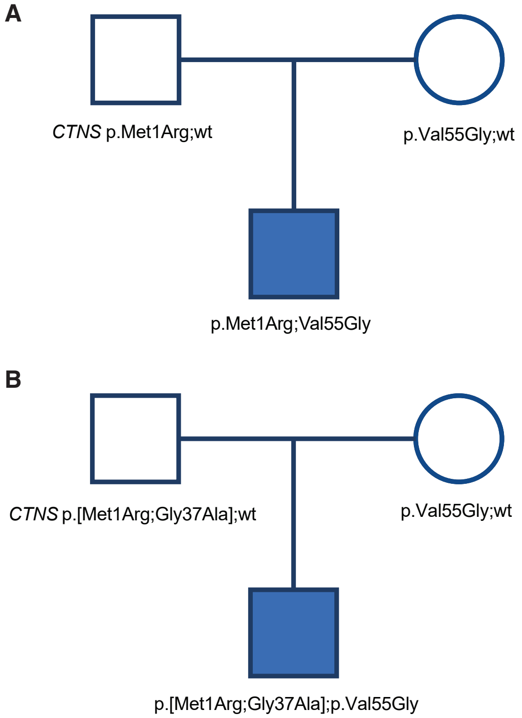

Figure 2. Cystinosis as an example for locus homogeneity and autosomal-recessive inheritance

(A) A simplex family affected with a child with the cystinosis phenotype and molecular genetic testing results for the *CTNS* gene. The affected offspring has typical cystinosis, which is associated with a nearly 96% yield of detecting two causative variants in *trans* using common sequencing methods that target exons and flanking introns.[10] There are no other genes known to be associated with this phenotype. In this family, *CTNS* was sequenced, and two novel missense variants were found in the proband and one of them in each parent. We estimate that there is at least a 95% likelihood that those are the causative variant—other possible mechanisms are implausible. Back-calculating the Bayesian points from the posterior probability of 95% in Table 2 shows that each allele would warrant +7.0 points. However, we limited this evidence type to +5.0 points (see text) for each allele, and because there was only one variant on each allele, each of these variants accrues the full +5.0 points. Were there to be additional affected children born to these parents, co-segregation pathogenicity evidence (PP1) would not be garnered because co-segregation cannot be considered evidence in traits with locus homogeneity.

(B) A second simplex family with the same scenario as in Figure 2A except that the paternal allele harbors two missense variants. In this case, the data suggest that the two paternally inherited variants in *cis* are equally likely to be the causative variant on that allele. Therefore, each of them has a 48% likelihood of being pathogenic. Referring to Table 2, that leads to assigning +2.5 points to each variant on that allele. The missense variant on the maternal allele would garner +5.0 points, as in Figure 2A. One should not divide the points arithmetically—that would be mathematically incorrect. One needs to refer to Table 2 each time, as further examples will show.

in *cis* in that allele of that gene. However, these recommendations occur in the context of the larger, Richards et al.[1] framework, which was focused on single-gene, Mendelian disorders of reasonably high penetrance. In this context, coding variants and exon-flanking intronic variants have a much higher prior probability of pathogenicity than do deeper intronic variants or variants 5′ or 3′ of the gene. We reasoned that the co-segregation evidence for coding and exon-flanking variants in *cis* within a single allele should be distributed across these relevant candidate variants. This division of evidence is determined by dividing the posterior probability of pathogenicity (not the points) of the *allele* equally among the relevant *variants* on the allele, then converting the resultant posterior probability to points and assigning these points to each variant (Figure 2B). For the *CTNS* example, each allele is nearly certain to be pathogenic given the approximately 96% diagnostic yield. If there are two variants on the allele, each variant has a probability of 48% to be pathogenic. In Table 2, the closest value to the posterior probability (column 1 of Table 2) of 48% (rounding down) is +2.5 points, which is assigned to each variant on that allele. This is demonstrated in the example in Figure 2B, and a worksheet for assessing this evidence is provided in Table S1.

We then recognized that evidence that is distributed among more than one variant in a single allele is fungible. In the hypothetical example shown in Figure 2B, the paternal allele was found to have two missense variants (p.Met1Arg and p.Gly37Ala), and the PP4 evidence was therefore equally distributed between both variants. If other evidence is available for these variants such that one variant is more likely to be pathogenic than the other, the PP4 evidence can be redistributed. In this hypothetical example, the p.Met1Arg variant would garner criterion PVS1_Mod (+2.0 points). The p.Gly37Ala variant would garner BP4 (−1.0 point) due to a low rare exome variant ensemble learner (REVEL) score[5,15] of 0.211 and, hypothetically, may have been observed in homozygosity in unaffected individuals (BS2, −4.0 points) and in *cis* with a known pathogenic variant (BP2, −1.0 point), giving a total of $-1.0 + -4.0 + -1.0 = -6.0$ points.[4] These new pieces of evidence shift the *relative* evidence distribution for the two variants from equal, 1:1 distribution to a difference of $+2 - -6 = +8$ points (odds of $81^{(8/6)} = 530{:}1$). Because this difference is greater than the +6-point (odds of $81^{(6/6)} = 81{:}1$) difference between the likely benign and likely pathogenic thresholds, the p.Met1Arg variant can be considered, for the purposes of the PP1/PP4/BS4 combined criteria, to be a single variant allele and can be assigned the full +5.0 points (limited by the allele cap). The p.Met1Arg variant would then garner +5.0 points for PP1/PP4/BS2 and +2.0 points for PVS1_Mod for a total of +7.0 points and a classification of likely pathogenic. The p.Gly37Ala variant can be classified using the BP4, BS2, and BP2 criteria as above, for −6.0 points, which is a classification of likely benign.

Integrating phenotype-specificity evidence and co-segregation evidence

Next, we moved to the more common scenario of locus heterogeneity. The simplest form of locus heterogeneity

Table 2. Conversion of diagnostic yield to Bayesian points

Diagnostic Yield	Points[a]
99.9%	12
99.8%	11.5
99.7%	11
99.6%	10.5
99.4%	10
99.2%	9.5
98.8%	9
98.3%	8.5
97.5%	8
96.5%	7.5
95.0%	7
93.0%	6.5
90.2%	6
86.4%	5.5
81.6%	5
75.4%	4.5
68.0%	4
59.6%	3.5
50.6%	3
41.5%	2.5
33.0%	2
25.4%	1.5
19.1%	1

"Diagnostic yield" is the historical demonstrated yield of pathogenic variants from molecular testing that is similar to the methodology used for the case under current analysis. It is wholly dependent upon the phenotype criteria matching as well. The conditional probability used for these calculations was $(81^{0.166})^{\text{Points}}$, where Points are the Bayesian points that are shorthand for the conditional odds, as specified in Tavtigian et al.,[4] where +1.0 is supporting, +2.0 is moderate, +4.0 is strong, and +8.0 is very strong evidence of pathogenicity. Fractional points have not been considered prior to this work in related papers.
In all cases, evidence is capped at +5.0 points for all locus evidence (PP1 and PP4) above +5.0 points per variant.
[a]These points apply to the allele, and if there is more than one variant on that allele, the evidence for the allele must be divided by the number of variants. See text.

has two unlinked causative genes. To construct realistic examples suitable for inductive reasoning, we chose the disorders *ERCC6*-related Cockayne syndrome and *ERCC8*-related Cockayne syndrome, which are both inherited in an autosomal-recessive pattern and caused by pathogenic variants in either *ERCC6* (MIM: 133540, located at chromosome 10q11.23) or *ERRC8* (MIM: 216400, located at chromosome 5q12.1). For this example, we assumed that we were trying to generate evidence to classify variants in *ERCC6*. About 70% of individuals with Cockayne syndrome have pathogenic variants in *ERCC6* and 30% in *ERCC8*.[16,17] For simplicity, we set aside phenotype variations in Cockayne syndrome and genotype-phenotype correlations with these two genes. As shown in Figure 3A, we considered an example of a hypothetical family of two affected children. In this scenario, we assumed that the family was tested for *ERCC6* only. There was no known consanguinity, but both parents were heterozygous for the p.Trp588Cys variant that was homozygous in each of their affected children and was the only variant identified in this gene. Again, the question arose as to whether there was co-segregation evidence that supported an assertion of pathogenicity for the p.Trp588Cys variant. Here, we concluded that indeed there was co-segregation evidence as there were no non-segregation events in the affected sibling. This observation provides $2^2/1$ or 4:1 odds in favor of pathogenicity (yielding an LOD score of approximately 0.6).

Given that the index affected individual was assumed to have typical Cockayne syndrome and the diagnostic yield of sequencing both genes in such cases is close to 100%,[18] the prior probability that a single detected *ERCC6* variant on each allele is the cause of the phenotype in this family was 70%. This prior is based on the concept of PP4 as outlined above, which is that if the testee has the phenotype, there is a 70% probability that there will be biallelic variants identified in that gene (and a 30% probability that the variants will instead be in *ERCC8*). The co-segregation evidence in this family comprised an additional conditional $\text{Odds}_{\text{path}}$ that favored the *ERCC6* locus, as that evidence was not likely to have arisen by chance alone under the alternative hypothesis that the causative locus was *ERCC8* (which, in this hypothetical case, was not sequenced). Because two variant alleles were discovered in *ERCC6*, a gene with a 70% contribution of the phenotype, the PP4 evidence for this locus would be +4.0 points (Table 2). To assess the evidence from co-segregation, we developed a lookup table that provides Bayes points values for common scenarios (Table 3). The additional affected sibling that shows co-segregation provides an additional +2.0 points (PP1, from Table 3) for a hypothetical total of +6.0 points, which would be capped at +5.0 points. In this example, +5.0 points would be applied to the p.Trp588Cys variant.

One can posit a scenario for Cockayne syndrome where two variants were detected in *trans* in *ERCC6* and no variants were detected in *ERCC8*. In this scenario, given that the combined diagnostic yield is essentially 100%, the absence of variants in *ERCC8* comprises not only evidence of benignity for *ERCC8* (because there are no variants) but also comprises evidence of pathogenicity for *ERCC6*. In this case, one could award PP4 points for the essentially 100% yield (in this scenario) for *ERCC6*, which would be capped at +5.0 points, and evaluation of co-segregation in this family would not be contributory.

We next modified the hypothetical case to demonstrate how to address compound heterozygosity in the testee. In Figure 3B, we show a similar scenario except that the parents harbor distinct variants. The father harbors the same variant as in the example above, but the mother harbors

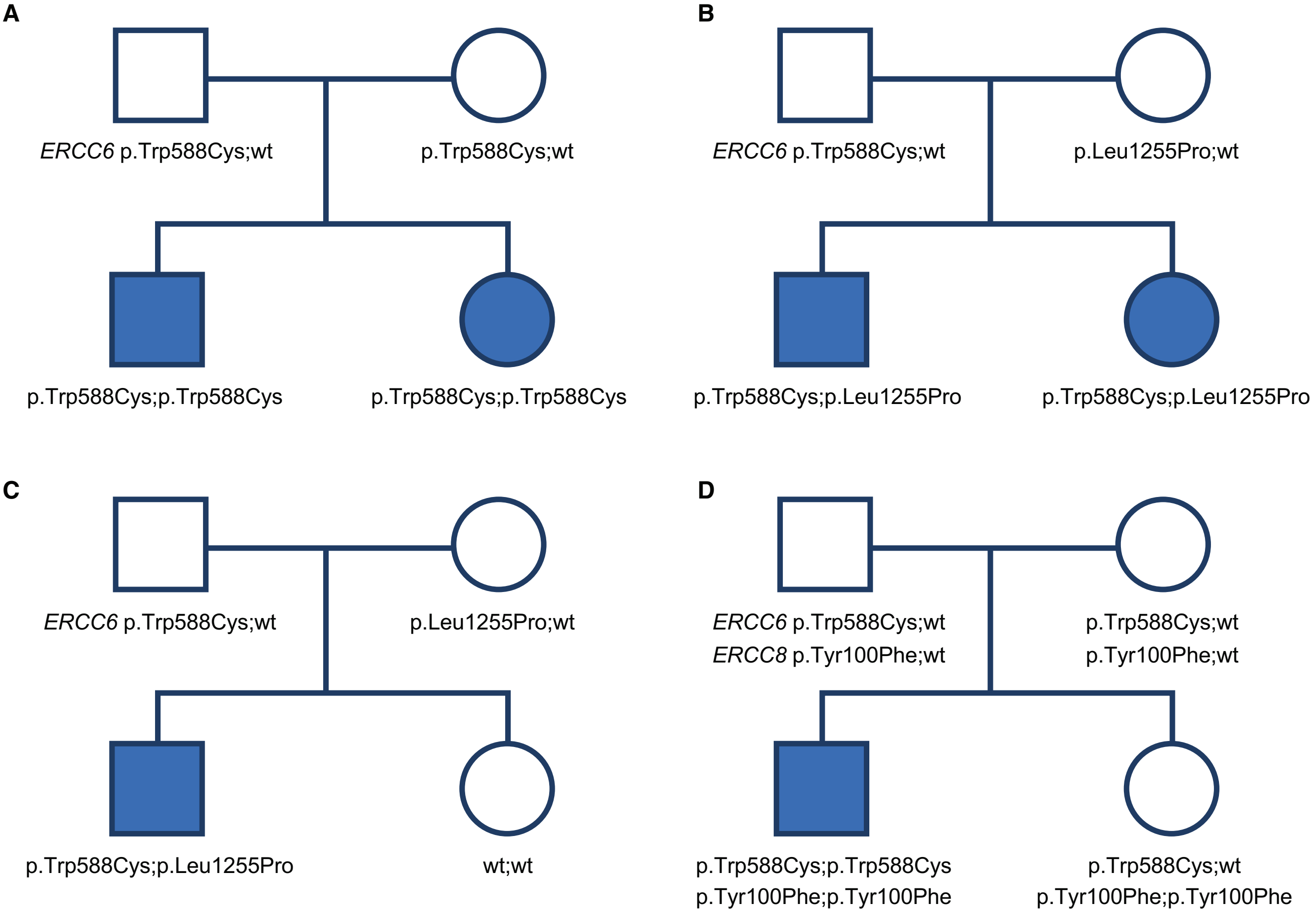

Figure 3. Cockayne syndrome as an example of locus heterogeneity and autosomal-recessive inheritance

(A) In this hypothetical example, a family with two children with the typical Cockayne syndrome phenotype have been sequenced for the more commonly mutated of the two genes associated with this trait, *ERCC6*. Both children are homozygous for a variant that is heterozygous in both parents. The novel variant is assigned +4.0 points, because the prior probability is 70%, given that 70% of patients with typical Cockayne syndrome have causative variants in *ERCC6*, and from Table 2, that corresponds to +4.0 points. However, because the evidence ceiling is +5.0 points, there is an affected sibling, and this trait exhibits locus heterogeneity, co-segregation evidence can be considered. Phase is established by individual II-1 and there is one co-segregating homozygous individual. Referring to Table 3, for a disorder with autosomal-recessive inheritance and homozygous genotype, each meiosis for an affected individual garners +2.0 points for the allele. These +2.0 points are then added to the +4.0 points, yielding +6.0 total points, which is reduced to +5.0 points because of the evidence cap.

(B) In this hypothetical example, a family with two children with the typical Cockayne syndrome phenotype have been sequenced for the more common of the two genes associated with this phenotype, *ERCC6*. Both children are compound heterozygotes for novel variants that are heterozygous in one of the parents. Both variants are assigned +4.0 points, because the prior probability is 70%, given that 70% of patients with typical Cockayne syndrome have causative variants in *ERCC6*, and from Table 2, that corresponds to +4.0 points. However, because the evidence ceiling is +5.0 points, there is a co-segregation in a sibling, and this trait exhibits locus heterogeneity, co-segregation evidence can be considered. Phase is established by individual II-1. Referring to Table 3, for a disorder with autosomal-recessive inheritance and compound heterozygosity, each co-segregating affected individual garners +2.0 point for each allele, which is added to the +4.0 points (PP4), summing to +6.0, which is reduced to +5.0 because of the cap. Note that less evidence is garnered here than in the example in Figure 3A, but the evidence was capped in that example.

(C) In another example using Cockayne syndrome, in this case the affected proband is compound heterozygous for a variant present in one each of his parents, and his unaffected sibling did not inherit the variants. As in Figure 3A, the PP4 evidence was +4.0 points. However, the co-segregation evidence is quite different than in the Figure 3A example because unaffected individuals provide less evidence of co-segregation than do affected individuals in autosomal-recessive inheritance. Referring to the second row of Table 3, one can see that one unaffected individual (who has an affected family member who is compound heterozygous) with consistent co-segregation garners +0.4 points for each allele, which is then rounded down to 0.0 points, which does not contribute to the evidence for pathogenicity of this variant. Were the family to have five unaffected children instead of one, that would garner +2.0 point (+0.4 × 5) for each variant.

(D) A family with one child affected with the typical Cockayne syndrome phenotype and testing results for the only two genes associated with this disease, namely *ERCC6* and *ERCC8*. Note that there is an observation of non-segregation with *ERCC8* in the unaffected sibling—she has the same genotype as the affected. This essentially excludes *ERCC8* and the associated variant from a causative role for this disorder in this family. In this case, the problem effectively transforms into a locus homogeneity problem with near 100% mutation sensitivity. In such a case, one would award +9.0 PP4 points plus +0.4 co-segregation points (for the unaffected), which is rounded down to +9.0 and then capped at +5.0 points.

a rare missense variant p.Leu1255Pro. In this example, the same points accrue to the two variants on the basis of the PP4 evidence (+4.0 points). As noted above in the *CTNS* example, the co-segregation evidence (PP1) is not divided across the two alleles so that both alleles (which harbor a single variant) are awarded +4.0 points. In this case, there is one affected sibling, which would garner a total of +2.0 additional points for a total of +6.0 points, which is capped at +5.0 points for each variant.

We next considered a scenario (Figure 3C) similar to the above except that the sibling is unaffected and did not inherit the same two variants as did the affected proband. In this case, there is the same PP4 evidence for the locus (as in the prior example) that would garner +4.0 points for each variant. However, in autosomal-recessive inheritance, unaffected individuals do not provide as much co-segregation evidence for the locus as do affected individuals. Looking at the second row of Table 3, one unaffected sibling would garner +0.4 points. Were there to have been five unaffected siblings with genotypes other than that of the proband, the co-segregation evidence would have garnered +2.0 points, which when added to the +4.0 points for the specific phenotype criterion would reach the maximum +6.0 points, which would be capped at +5.0 points.

Benign evidence at one locus can provide pathogenic evidence for another locus

The above three examples considered segregation data available at one of the two loci for Cockayne syndrome. Additional evidence could be gained by evaluating multiple loci simultaneously, as is commonly done in panel, exome, and genome testing. In the next hypothetical example (Figure 3D), we posited that the *ERCC6* variants segregated with disease in the two children (as in the example in Figure 3C), but there was also an *ERCC8* variant for which the parents were both heterozygous, and there was a non-segregation such that the unaffected sibling inherited the same two *ERCC8* variants as did the proband. This provides evidence against the hypothesis of causality of these *ERCC8* variants, and the negative co-segregation data at *ERCC8* essentially exclude this locus as causative in this family. Additionally, the *ERCC8* non-segregation provides indirect support for the pathogenicity of variants at the *ERCC6* locus, which is another example of the fungibility of evidence, in this case fungibility across loci for disorders with locus heterogeneity. In effect, this two-locus test scenario is reduced to a locus homogeneity scenario because if the likelihood of *ERCC8* causing the phenotype drops to zero, the likelihood for *ERCC6* causation rises to essentially 1 (i.e., 100%). Data at one locus providing indirect evidence about the pathogenicity of a variant at another locus is an evidence concept that was not considered in Richards et al.[1] The results from this testing outcome would be scored at +4.0 points for the *ERCC6* variant for PP4 and +0.4 points for the co-segregation evidence (PP1) in the unaffected sibling for a total of +4.4 points. However, as noted above, the non-segregation data at *ERCC8* essentially make this a locus homogeneity scenario, and the likelihood that an allele with a single variant on it is causative is ~100% (not 70%, as was the case when both loci were being considered). Referring to Table 2, we estimate that this value is practically 99%, which corresponds to +9.0 points, but this is reduced to +5.0 points due to the evidence cap. Thus, the non-segregation data at *ERCC8* provided an indirect +1.0-point increment of evidence for the *ERCC6* variant, again limited by the cap.

For disorders with autosomal-recessive inheritance and a high degree of locus heterogeneity, the PP4 concept should be used with caution. For example, the Bardet-Biedl syndrome phenotype (MIM: 209900) is associated with >27 loci, and while *BBS1* (MIM: 209901) is the most commonly involved locus at 23%, there is likely to be substantial variation in this distribution across populations.[19] One could refer to Table 2 and suggest that it would be appropriate to assign +1.0 point for the finding of two variants in *trans* in *BBS1* in a typically affected individual, but this assumption may be subject to error given variation in diagnostic yields across populations. Nonsyndromic hearing loss with autosomal-recessive inheritance poses a more extreme example with at least 66 associated genes.[20] In both scenarios, it is prudent to only assign evidence points using the PP1/BS4 co-segregation criteria (Table 3) rather than using the lower ranges of positive evidence in Table 2 for the specific phenotype (PP4). We recommend a lower limit of +1.0 points for PP4, which would be equivalent to a 20% diagnostic yield. However, as with the *ERCC6*/*ERCC8* example above, multigene testing that exhaustively excludes plausible variants in other known disease genes can provide indirect evidence, implicating the single locus in which candidate variants are identified.

Adapting these principles to autosomal-dominant and X-linked inheritance

Evaluating this evidence for disorders with autosomal-dominant inheritance is in most respects simpler than for autosomal recessive; therefore, the reasoning process described above will not be developed here in detail for autosomal-dominant inheritance. However, there are a few nuances of testing in disorders with autosomal-dominant inheritance that are worth addressing. We selected *FBN1*-related Marfan syndrome (MIM: 154700) as an example. For persons with the typical Marfan syndrome phenotype (fulfilling the clinical criteria according to the Ghent nosology), 91%–93% will be found to have a causative variant in *FBN1*.[21] No individual with typical Marfan syndrome has been found to have a causative variant in another gene. Although Marfan syndrome has locus homogeneity, it is a distinct situation from that of cystinosis. Because variants in *trans* to the causative variant are typically not causative (in contrast to the autosomal-recessive case, where both alleles are always pathogenic in an affected), for two variants detected in a single tested

Table 3. Points derived for co-segregations for traits

		Number of individuals with co-segregations				
		1	2	3	4	5
Autosomal-recessive affected	Points[b,c]	2.0	4.0	6.0	8.0	10.0
Autosomal-recessive unaffected[a]		0.4	0.8	1.2	1.6	2.0[d]
Autosomal-dominant affected and unaffected[a]		1.0	2.0	3.0	4.0	5.0
X-linked-recessive male affected and unaffected[e]		1.0	2.0	3.0	4.0	5.0

[a]Only count unaffected individuals if disease is fully penetrant. Do not count unaffected parents as they are used to establish phase.
[b]These points apply to the allele, and if there is more than one variant on that allele, the evidence for the allele must be divided by the number of variants. See text.
[c]Capped at +5.0 points for all locus evidence (PP1 and PP4) above +5.0 points per allele.
[d]Continue to add +0.4 points for each meiosis above five.
[e]Additional segregations can be counted for obligate heterozygous females.

individual where there are two alleles, each of which could harbor a pathogenic variant (no parent or child tested), each variant should be assigned a lower-weighted PP4 criterion than in the *CTNS*-related cystinosis example above. A single parent-to-child meiosis, in which both parent and child are affected, can be used to distinguish the co-segregating allele (Figure 4A). This would constitute strong evidence against pathogenicity for the variant inherited from the unaffected parent. Once phase has been established between the variant and phenotype with a single meiosis, further co-segregations with the phenotype for a disease with locus homogeneity and high diagnostic yield does not constitute any additional increment of evidence for pathogenicity (reasoning that intragenic crossovers are too rare to be considered) based on the same reasoning above regarding cystinosis and *CTNS* co-segregation. At this point, the locus homogeneity autosomal-dominant inheritance situation parallels the autosomal-recessive situation above in that co-segregation (PP1) is no longer useful, but the specific phenotype criterion (PP4) would apply as +6.0 points (from Table 2) capped at +5.0 points.

We emphasize that the phenotype-specificity points are dependent upon the degree to which the phenotype points to one, and only one, locus. The pleiotropic nature and the specificity of the combination of the findings that leads to a high Ghent score generates that specificity for *FBN1* variants. That phenotype specificity would not pertain to an individual who presents with isolated thoracic aortic aneurysm, nor would it pertain to an individual who was incompletely phenotyped, nor to a young child. Thoracic aortic aneurysm, as an isolated finding, exhibits locus heterogeneity and lower diagnostic yield, and therefore lower PP4 points (or perhaps none) would be more appropriate. Young children typically have few specific manifestations that would support the locus homogeneity argument. Instead, co-segregation evidence could accrue to that variant based on that phenotype co-segregating in a family.

Co-segregation evidence can be very useful for a disorder with autosomal-dominant inheritance that has been associated with more than one locus. Tuberous sclerosis complex (MIM: 191100 and MIM: 613254) is an excellent example as it has two loci, with ~26% attributable to *TSC1* (MIM: 605284), ~69% attributable to *TSC2* (MIM: 191092), and ~5% with no definitive attribution.[22] In familial cases, it is common to perform panel testing such that both *TSC1* and *TSC2* are interrogated simultaneously (Figure 4B). In such cases, it is common to detect variants in both genes, although only one of the two genes is likely to co-segregate with the phenotype in a given family. In the example in Figure 4B, the recombinants observed at one locus, *TSC2*, provide indirect evidence in support of the pathogenicity for a variant in *TSC1*, which segregates with the phenotype. With the two non-segregations (II-2 and II-3), *TSC2* is essentially excluded as a candidate. Analogous to the Cockayne syndrome example in Figure 3D, this effectively converts this tuberous sclerosis testing scenario into one of locus homogeneity—because *TSC2* is excluded, ~95% of the time, a causative variant will be identified in *TSC1*. Therefore, the phenotype-specificity (PP4) points can be allocated to *TSC1* (+7.0 points) and two non-recombinants (PP1; +2 points) for a total of +9.0 points, which would be capped at +5.0 points, before other evidence was added. Disorders with autosomal-dominant inheritance and higher orders of locus heterogeneity should be handled similarly to the autosomal-recessive examples above.

Reduced penetrance is a common attribute of disorders with autosomal-dominant inheritance. It would not be practical to develop simple advice for counting evidence for unaffected individuals in families with such disorders—there are too many variables. This can be handled by using an affected-only approach and assessing co-segregation in only those individuals using the points in Table 3. Of course, some information is lost when doing so, and there is a risk of ignoring evidence against pathogenicity in families with unaffected heterozygotes (in autosomal-dominant inheritance), but this can only be remedied with formal linkage analysis that accounts for disease penetrance and ages of family members (i.e., liability classes). We recommend only assessing segregations in affected individuals according to Table 3 and only applying PP4 evidence

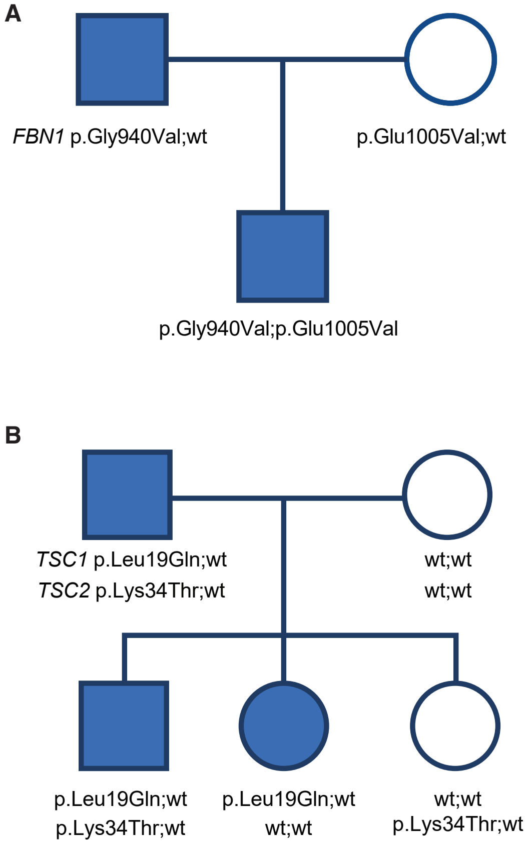

Figure 4. Examples of autosomal-dominant inheritance; Marfan syndrome for locus homogeneity and tuberous sclerosis for locus heterogeneity
(A) An example of the use of a single meiosis to set phase in a disorder with autosomal-dominant inheritance. Using the approach described here, because the yield is 91%, the likelihood for each variant is 45.5%, which would correspond to +2.5 PP4 phenotype-specificity points (Table 2) would be assigned to each variant in the child had the parents not been tested. However, one can set phase by observing that the p.Gly940Val variant is co-segregating with the phenotype. Thus, the only remaining candidate variant in the child is p.Gly940Val, and therefore the full 91% yield now pertains, which would correspond to +6.0 points (Table 2) but is capped at +5.0 PP4 phenotype-specificity points, which can be assigned to that variant.
(B) An example of autosomal-dominant inheritance of tuberous sclerosis (TS). In this family of three affected individuals, a single variant has been identified in both *TSC1* and *TSC2*. Because 26% of TS is associated with *TSC1* and 69% with *TSC2*, the p.Leu19Gln *TSC1* variant is assigned +1.5 points (rounding down to 25% in Table 2), and the p.Lys34Thr *TSC2* variant is assigned +4.0 points (rounding down to 65% in Table 2) for phenotype-specificity points (PP4). The *TSC1* variant can be phased using individuals I-1 and II-1. The *TSC1* variant also garners +2.0 points for the co-segregations (II-2 and II-3) for a total of +3.5 points. However, the *TSC2* variant shows non-segregations in both II-2 and II-3, effectively excluding that variant (and that gene) from being causative in this family. Therefore, the PP4 points need to be reevaluated. Now, the expected yield for *TSC1* would be more than 95%, and thus +7.0 points (points from 95% on Table 2) would be awarded to the *TSC1* variant, which would also garner +2.0 additional points for the co-segregations but be capped at +5.0 points irrespective of those segregations.

points if the diagnostic yield for the gene under consideration is over 20%, assuming variants are identified in more than one gene (applying 1.0 points or above). Examples of phenotypes for which PP4 would be inappropriate are many but include arrhythmia, intellectual disability, and seizures. Figure 5 demonstrates an example of a disorder, dilated cardiomyopathy, with diagnostic yield below 20%, reduced penetrance, and a pedigree with incomplete genotype information. Some disorders have the attribute of measurable, or in some cases substantial, phenocopy rates, e.g., colon cancer. We do not recommend our heuristic approach for such traits as valid co-segregation evidence can only be generated with formal segregation analysis.

X-linked recessive inheritance is similar to autosomal dominant in that consistent co-segregations for an affected or unaffected male would garner +1.0 point (as most are highly penetrant and have low phenocopy rates). Co-segregation of a variant in an unaffected female can also count for +1.0 point if, for example, she has an affected brother or son. This is shown by the example in Figure 6.

The considerations for locus heterogeneity can be generalized to disorders for which there is a substantial fraction of occurrences of the phenotype where the employed testing method does not identify a causative variant (disorders with diagnostic yield ≤90%). In these cases, co-segregation evidence can be generated for the known locus or loci, with PP4 points awarded as per Table 2 and the fraction of negative cases (=1−diagnostic yield) can be treated as an unknown or phantom locus, the same as the *ERCC8* locus was treated in Figure 4, which is as an untyped locus.

The prior examples all focused on relatively simple scenarios where the phenotype-specificity and co-segregation evidence used as $Odds_{path}$ were all garnered from the tested family. However, one can add evidence from other families, whether they are historical cases from the testing laboratory or found in publications. We recommend considering the individual whose phenotype is most specific for the relevant genes, but they should only be used once. The number of PP4 points to apply is based on the diagnostic yield of the given gene for individuals with the exact phenotype constellation upon which the diagnostic yield data were derived. For example, if the testee is a newborn with hearing loss and a homozygous *SLC26A4* variant (MIM: 605646) but no temporal bone imaging has been performed, but there is another case in the literature of a child with hearing loss and enlarged vestibular aqueducts of the inner ear who has the same variant in the *SLC26A4* gene for Pendred syndrome (MIM: 274600), +2.5 points for PP4 can be awarded for the published case based on the 50% diagnostic yield for children with hearing loss and this inner ear finding.[23] If a published case is used for the PP4 criterion component of this assessment, then the testee should not be used for the PP4 criterion. Other cases should be counted under the PS4 or PM3 criteria for case:control or case-counting assessment evidence. If the disorder has locus heterogeneity and co-segregation evidence will be used, all known families harboring the same variant can be used. If there are

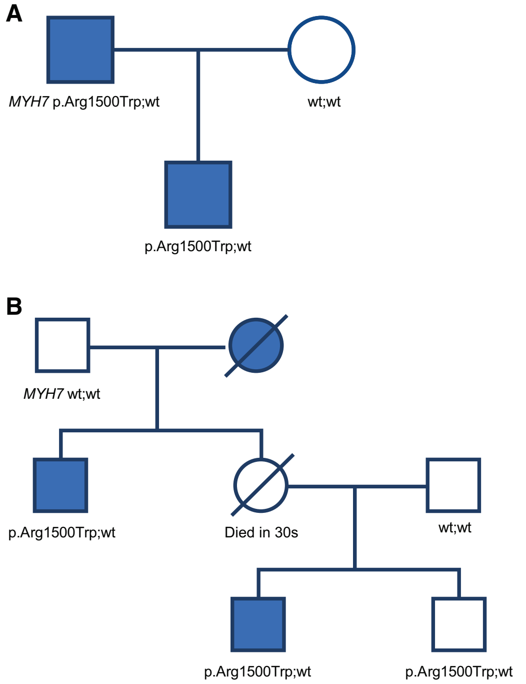

Figure 5. Example of autosomal-dominant inheritance with low diagnostic yield—dilated cardiomyopathy (DCM)
For this disease, diagnostic yield for *MYH7* does not reach 20%, so PP4 cannot be applied. One can eliminate the need to set phase by observing no variation on the second allele.
(A) The only candidate variant in the proband (II-1) is p.Arg1500Trp, and +1.0 point from Table 3 can be awarded for the single co-segregation from the affected father (I-1).
(B) In this family there are at least two co-segregations (II-2 [obligate] and III-1), which garners +2.0 points from Table 3. One could also justify counting individual I-2 given that a positive genotype can be inferred from the genotype-negative I-1 individual, adding another point for a total of +3.0 points. The absence of phenotype in individual III-2 who harbors the variant is not considered given reduced penetrance and age-related onset of DCM.

multiple alleles at a given locus (e.g., Figure 4A), one should always use the first meiosis (two for autosomal recessive) to establish phase within each family. If there is only a single allele under consideration, setting phase is not needed (e.g., Figure 5A). Co-segregations are then counted using the point values in Table 3, summing across families, and capped at +5.0 points if the allele has a single variant. If there is more than one variant on the allele, evidence points (which may exceed the +5.0 threshold) should then be divided among the plausible candidate variants, as demonstrated in the prior example in Figure 3, and then the divided evidence should be capped at +5.0 per variant.

In this paper, we have applied the points-based Bayesian approach[4] for summing evidence across evidence types to arrive at a final variant classification. If the laboratory is using the previously described six-level evidence scheme of Richards et al.[1] (pathogenic very strong, strong, moderate, and supporting and benign strong and supporting), then the point values can be converted to those descriptive levels using Table 4. We recognize that most curation platforms do not accommodate points levels. To adapt to this, users may consider allocating the combined PP1/PP4 points-based evidence resulting from the recommendations herein across the PP1 and PP4 criteria. For example, if the variant warrants +5.0 points of evidence, the analyst could allocate that to PP1_Strong and PP4_Supporting.

Summary of this heuristic approach to simple locus and co-segregation evidence

1. To evaluate a variant using locus-specificity (PP4) and co-segregation (PP1, BS4) evidence, first gather relevant information on the gene-phenotype dyad(s).
   a. Obtain the best available information on the locus homogeneity/heterogeneity, the specificity of the phenotype, the expected diagnostic yield of the testing methodology used, the test results of the current testee and family, and previous literature reports of the relevant variant.
2. Determine whether the presence of the phenotype is substantially specific to support a determination of locus homogeneity with high diagnostic yield (above 90%) vs. heterogeneity.
   a. If the trait has locus homogeneity…
      i. assign the phenotype-specificity (PP4) points as defined in Table 2, estimating the points from the appropriate posterior probability.
      ii. do not use segregation analysis to support the pathogenicity.
      iii. distribute the evidence among the variants on the implicated allele.
      iv. evaluate other forms of evidence.
      v. and if other forms of evidence provide much stronger evidence for one variant over others on the allele, redistribute the evidence to the more pathogenic variant(s) on the allele.
      vi. cap this evidence at +5.0 points.
   b. If the trait has locus heterogeneity or locus homogeneity but the diagnostic yield is low, suggesting other causal genes may exist…
      i. assign the phenotype-specificity PP4 points, using Table 2 and aggregating across the relevant loci, according to best available data on the gene-phenotype dyad and expected diagnostic yield for the testing methodology that is being used.
      ii. add co-segregation points from the current tested family and literature cases according to Table 3.
      iii. reassess the phenotype-specificity points if non-segregation (or comprehensive coding sequence evaluation for gene-phenotype dyads that are known to have a gain-of-function mechanism) excludes one or more loci.
      iv. and if there is more than one variant on the allele, and other forms of evidence provide much stronger evidence for one variant over others on the allele, redistribute the evidence to the more pathogenic variant(s) on the allele.
      v. cap at +5.0 points.

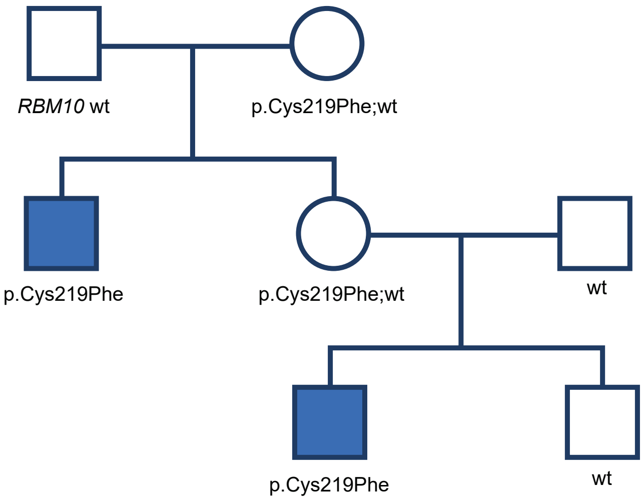

Figure 6. An example of co-segregation in a three-generation family with X-linked-recessive inheritance
The affected males have typical TARP syndrome (talipes equinovarus, atrial septal defect, Robin sequence [micrognathia, cleft palate, and glossoptosis]) and persistent left superior vena cava, (MIM: 311900), which is only known to be associated with the *RBM10* gene. The variant identified in this family is p.Cys219Phe. The diagnostic yield is not known for this phenotype because it is so rare, so PP4 points are not awarded. Phase is established in an affected male in disorders with X-linked inheritance without the need for observation of any meioses. In this family there are four co-segregations (II-1, II-2, III-1, and III-2), which garners +4.0 points from Table 3. Note that individual II-2 garners +1.0 point because her status as a carrier is affirmed by having an affected brother and son.

3. If there is low penetrance, a high phenocopy rate, conflicting co-segregation information, or a very large pedigree, use formal linkage analysis.

Discussion

At least four groups have published studies on how to use co-segregation evidence to classify variant pathogenicity.[7,24–26] Two of the papers focused on examples of autosomal-dominant inheritance in cancer syndromes,[7,26] one focused on *ENG*-related hereditary hemorrhagic telangiectasia and *ACVRL1*-related hereditary hemorrhagic telangiectasia (MIM: 187300 and MIM: 600376),[24] and a fourth was disease agnostic but limited to autosomal-dominant inheritance.[25] In particular, the papers addressing cancer syndromes raised the issue of incomplete and age-dependent penetrance for which Thompson et al.[7] proposed to use penetrance classes and Mohammadi et al.[26] proposed use of a continuous function of age-of-onset only, arguing that other variables affecting penetrance were too difficult to determine in the practice of clinical genetic testing. In general, all four papers proposed to evaluate the co-segregation evidence for a variant by a likelihood ratio calculation, possibly with some simplifications. Importantly for our work, the evidence evaluated in those four methods only concerned the variant under evaluation and nearby variants—none of them considered evidence for or against linkage at other loci, which can enable additional evidence to be applied and improve the ability to attain a pathogenic classification. Prior publications on co-segregation evidence for variant classification did not include scenarios in which multiple, possibly associated genes were tested in parallel and non-segregating variants were observed in at least two genes. Exclusion at one possible locus was not treated as positive evidence for a second locus, particularly for diseases with only a handful of associated genes or when co-segregation analysis excludes many of the candidate genes and only one or a few genes remained. Originally, the LOD score 3.0 criterion for autosomal genetic linkage analysis was developed by Cedric Smith, Newton Morton, and others as a stopping criterion in a framework where loci were tested sequentially, so the number of excluded loci was not considered.[8]

Importantly, we recognized that co-segregation evidence is inextricably related to the Richards et al.[1] phenotype-specificity criterion (PP4), which specified that "patient's phenotype or family history is highly specific for a disease with a single genetic etiology." We have determined that in the case of disorders with locus homogeneity and high diagnostic yield, the PP4 evidence criterion can be applied at point values significantly higher than supporting. However, in these cases, co-segregation evidence cannot be applied given the overlap in the underlying evidence for implicating the locus. Further, we enable PP4, in conjunction with PP1, to be applied in diseases with locus heterogeneity based on the known diagnostic yield for a given gene-phenotype dyad,[27] allowing PP4 evidence to increase as other loci are excluded.

A crucial component of our reasoning for the evidence scaling and the use of the phenotype-specificity criterion (PP4) is the availability of robust data on diagnostic yield based on a clearly articulated definition of the phenotype. We recognize that there can be an element of circularity or confirmation bias in this. Hypothetically, if a disorder is incorrectly assumed to have locus homogeneity, then benign variants in that gene may be misclassified as pathogenic, which appears to further support the locus homogeneity, and variants at other loci may never be considered. The strongest evidence to exclude an incorrect assertion of locus homogeneity would be an observation of non-segregation of variants at that locus with the phenotype. To our knowledge, that has never been observed in the examples we have selected in this paper (cystinosis and Marfan syndrome), which suggests that the assertion of homogeneity for those disorders is extremely unlikely to be falsely established because of circularity. That being said, this form of evidence should be used only in cases where the clinical data are robust, whether it be the phenotype information for a clinical test submission or the data in a scientific manuscript. If individuals are incompletely phenotyped, other loci must be considered. For many rare disorders, the clinical and

Table 4. Conversion of combined phenotype specificity and co-segregation Bayesian points to a combined PP1/PP4 strength

Points	0–0.9	1–1.9	2–2.9	3–3.9	4–4.9	≥5
Maximum allowable strength of combined codes	not applicable	PP1_Supporting or PP4_Supporting	PP1_Moderate or PP4_Moderate or PP1_Supporting + PP4_Supporting	PP1_Supporting + PP4_Moderate or PP4_Supporting + PP1_Moderate	PP1_Strong or PP4_Strong or PP1_Moderate + PP4_Moderate	PP1_Strong + PP4_Supporting or PP4_Strong + PP1_Supporting

genetic evidence is overall sparse, and an assertion of locus homogeneity should be viewed with some skepticism. In such cases, the laboratory should err on the side of downgrading the evidence strength or not use the PP4 criterion to avoid potentially overestimating this evidence. As noted in the *FBN1*-related Marfan syndrome example above, the locus-specificity (PP4) evidence was based on the pleiotropic nature of the phenotype, that the manifestations are individually uncommon or rare, and that having multiple rare manifestations in distinct organ systems is unlikely to occur by chance alone. It is critical to distinguish locus heterogeneity from a scenario of an inadequate evaluation of an individual. These guidelines would support the approach of awarding PP4 phenotype-specificity points for an *FBN1* variant in an individual who was thoroughly evaluated and meets the Ghent criteria. Although the endophenotype of thoracic aortic aneurysm is present in many individuals with pathogenic variants in *FBN1*, that phenotypic manifestation alone is not a specific predictor for pathogenic variants in *FBN1*, being associated with at least five other loci. Thoracic aortic aneurysm has locus heterogeneity, but classic Marfan syndrome has locus homogeneity. Only variants from individuals who have undergone thorough evaluations and who meet Ghent criteria should be awarded PP4 points. Individuals who are inadequately evaluated or are too young to have developed the more specific manifestations of Marfan syndrome should not be awarded PP4 phenotype-specificity points. We recognize that clinical testing laboratories may not have access to the level of phenotypic data necessary to make this determination, especially from a test submission form. In these cases, the laboratory should work with the ordering clinician to gather the necessary evidence to make this assessment and only score the PP4 evidence when it is justified and supported. In these cases, co-segregation evidence would be more appropriate to use rather than the specific phenotype evidence. Alternatively, if the same variant has been reported in the literature and that report has robust phenotype data, those data can be used for PP4 instead of that of a testee with testing submission with sparse data.

Published reports of affected individuals can be used to establish PP4 evidence for a variant (as opposed to the current testee). This necessarily raises the question of the overlap and distinction of PP4 evidence vs. PS4 case-counting evidence. Full consideration of the issue of PP4 and PS4 overlap is beyond the scope of the present work. A possible approach might be to add case-counting (PS4) data to the PP1/PP4 data but cap that at a level that would not allow PP1/PP4 and PS4 together to exceed the high end of likely pathogenic (+9.0 points) analogously to how we have capped PP1/PP4 data alone to not exceed the high variant of unknown significance (VUS) level (+5.0 points). Analysts should use their professional judgment to allocate evidence between PP4 and PS4, applying it to responsibly come to a reasonable conclusion. It is worth noting that a previously observed family can count as either PP4 or PS4 evidence but not both. In addition to counting a single individual as one of those two forms of evidence, the co-segregation data from family members of that individual can count as additional PP1 evidence. There will be a challenge of keeping track of where evidence from a given observation is applied (PP4 vs. PS4). In the future, we hope to provide more specific advice on many of these issues and questions.

We have added a concept to the Richards et al. framework, which is that when a phenotype has locus heterogeneity, evidence that variants in one or more loci are not segregating with a trait increases the likelihood that variants at co-segregating loci are pathogenic (PP1 evidence). Similarly, evidence of absence of variants at one locus can increase the diagnostic yield points awarded to another locus (PP4 evidence). That is, non-segregation or phenotype-specificity exclusion of one locus increases the likelihood of pathogenicity of variants at other loci that co-segregate with the phenotype. The consideration of excluded loci is important now that multiple candidate genes are routinely tested in parallel by gene panels, exomes, and genomes. Formalizing the role of excluded loci allows our framework to quantify the intuitive notion that negative evidence for pathogenicity at one locus is indirect, positive evidence for pathogenicity at other associated loci. Typically, it takes a number of years after the discovery of the molecular etiology of a disorder to accumulate sufficient testing experience in the scientific literature and in diagnostic laboratories to make this assessment. A good source of this information is the mutational yield tables in GeneReviews entries. When such data are not available, this evidence concept should not be implemented. We have described this as one form of evidence fungibility. Moreover, formalizing the role of excluded loci is helpful to justify the intuition that if two clinical tests find the same single variant at one locus using nested panels with different numbers of genes, then the testing panel with more genes known to contribute to diagnostic yield (and hence more excluded loci) yields more evidence, even though both tests find the same variant.

When co-segregation data are applied to variant classification (as opposed to disease gene mapping), it is important to consider this nuance as exclusion by non-segregation at one (or more) loci can substantially increase the pathogenicity of variants at linked loci. In autosomal-dominant inheritance, autosomal-recessive inheritance with homozygosity, and X-linked inheritance, non-segregations do provide evidence of benignity, which was conceptually captured by the BS4 criterion. In these cases, we recommend assigning −4.0 points to such variants for these observations. We have not formally quantified this evidence strength but are recommending continuation of this evidence strength from Richards et al.[1] However, it is critical to recognize that in autosomal-recessive inheritance with compound heterozygosity in the affected proband, the existence of non-segregations in relatives at a given locus provides little to no evidence that those variants are not pathogenic. This is because only one of the two alleles is necessarily benign, and the non-segregation data cannot distinguish in a single family which of the variants is benign.

Many Mendelian disorders have attributes that are amenable to heuristics that simplify the assessment of this type of evidence. Many have high penetrance and low phenocopy rates. Many phenotypes are associated with few, or in a number of disorders, only one gene. By exploiting these attributes and coupling this to the formal Bayesian framework, we have developed a relatively simple heuristic that allows for a points-based approach to the specific phenotype (PP4) and co-segregation (PP1/BS4) criteria. The general issue of the validity of co-segregation data for assessing pathogenicity of variants derives from the fact, as stated in Richards et al., that "… segregation of a particular variant with a phenotype in a family is evidence for linkage of the locus to the disorder but not evidence of the pathogenicity of the variant itself."[1] More specifically, co-segregation evidence supports the pathogenicity of all variants that are in *cis* with, and do not recombine with, the detected variant that is used to assess co-segregation. Importantly, this includes detected and undetected variants, which is why we capped these two forms of evidence (PP1 and PP4) at +5.0 points. With respect to the pathogenicity of alleles that include more than one variant, it is possible that each variant is benign when present alone or that both variants are independently pathogenic. Our recommendations do not take this into account, and while this has been observed,[28] we assume that it is rare. Future work should evaluate this more thoroughly and consider approaches to this issue if it turns out to be frequent or impactful.

We have developed a heuristic that allows evidence to be apportioned among variants on alleles that have been shown to be associated with the phenotype, either by the specific phenotype (PP4) concept or by co-segregation (PP1/BS4). For scenarios where the data are limited, we provide a heuristic that allows the data to be used in a simple manner that avoids overestimating the strength of the evidence. A key contributor to this concept of the fungibility of evidence among variants on a single allele is that current technologies nearly universally interrogate the entire coding sequence and flanking introns of a gene. In years past, only parts of genes or even selected variants (so-called "hot spots") may have been interrogated in the testing assay, which limited the ability to assess the likelihood of the presence of other candidate variants on that allele. Current methods, especially genome sequencing, substantially eliminate this issue.

Where data are rich and extensive or the disorders have complex attributes such as low penetrance and a high phenocopy rate, the only useful and valid method is a formal maximal likelihood estimation, which requires statistical genetics expertise (e.g., http://bjfenglab.org/). We have elucidated a heuristic for the simpler scenarios in this paper, but detailed guidance on the formal analyses is beyond the scope of this guidance. That being said, we have made several key observations that should be taken into account when performing these formal maximum likelihood analyses to inform variant classification. First, our recognition that the PP1 and PP4 criteria are related should be taken into account and not double counted. Second, our recognition that evidence is fungible among variants on a given allele should be integrated into formal segregation analyses. Third, formal methods should be developed for the fungibility of evidence across multiple non-linked loci when traits have locus heterogeneity and evidence is available from more than one of the loci.

We note that we have fitted this analysis to the Bayesian framework and its points adaptation, which assumed a prior probability of pathogenicity of 0.102[4] (originally 0.10[2]) and a conditional $\text{Odds}_{\text{path}}$ for supporting evidence of 2.08:1. In the course of exploring co-segregation evidence, we have recognized that the conditional $\text{Odds}_{\text{path}}$ could instead be 2.0:1 being based on the foundational biology of genetics, which is that of meiotic segregation. Although it is beyond the scope of these recommendations, we suggest that the Bayesian system be shifted to a basis where the $\text{Odds}_{\text{path}}$ of a supporting piece of evidence be 2.0:1 and the prior probability be adjusted to 0.125. This would put quantitative variant classification on a firm theoretical foundation based on first principles.

The previous four publications on the co-segregation criteria for pathogenicity did not consider autosomal-recessive or X-linked inheritance.[20–23] The Richards et al. committee intended co-segregation evidence to apply to all modes of inheritance (H. Rehm, personal communication). We developed our reasoning primarily using disorders with autosomal-recessive inheritance to remedy this issue. In fact, it is these well-chosen disorders with autosomal-recessive inheritance that clearly exemplify the concept that for locus homogeneity, one cannot generate co-segregation evidence beyond the phenotype-specificity evidence already captured.

It is also important to note that for some phenotypes, such as Ellis-Van Creveld syndrome, there are pairs of genes associated with these gene-phenotype dyads that are in tight linkage with each other (*EVC* and *EVC2* on chromosome 4 [MIM: 604831 and MIM: 607261])[29] so that co-segregation evidence for a locus can include variants in both the linked genes. In these cases, evidence must be distributed among the two loci in the same manner that evidence must be distributed across two variants in a single gene without other distinguishing evidence for pathogenicity.

We are using a relatively high degree of precision in this paper, with the Bayesian points being considered to four-tenths of a point for co-segregation data. This is a level of precision that reflects the inherent precision of co-segregation calculations, which is in sharp contrast to most other forms of evidence used to classify variants. While we have used that level of precision for co-segregation evidence (apparent in the autosomal-recessive compound heterozygous examples), we only recommend using specific phenotype points scores in increments of 0.5 points, and this should only be done when those attributes of the disorder are robustly established. In many rare disorders, such precise data may not be available and such evidence points should be applied with caution, erring on the side of less evidence strength and rounding down the strength of evidence when it falls between two values.

In summary, we have developed a heuristic for assessing the specific phenotype (PP4) and co-segregation (PP1 and BS4) criteria that support a point-counting approach to the Bayesian adaptation of the ACMG/AMP criteria that quantitatively assesses these forms of evidence. This heuristic can be used with disorders that have specific phenotypes and a quantified molecular testing yield and can be adapted to disorders with reduced penetrance. Formal segregation analysis is still needed for disorders with a significant phenocopy rate and larger families with more complex pedigrees, such as consanguinity. This work is part of an overall effort to evolve ACMG/AMP variant classification from its former basis of primarily expert opinion into an objective, quantifiable, scientifically based method.

Supplemental information

Supplemental information can be found online at https://doi.org/10.1016/j.ajhg.2023.11.009.

Acknowledgments

This SVI subgroup is indebted to the parent ClinGen SVI group and ClinGen leadership for supporting, facilitating, and critiquing this work as it evolved in the subgroup. We are grateful to Marwa Elnagheeb and Ryan Webb for assisting with the gene-disease curations that were needed for this work. All the pedigrees in this manuscript were hypothetical and fictional. None of the evidence described in this paper should contribute to the actual classification of any of the variants we have used to illustrate the concepts. The authors acknowledge Drs. Margaret Adam and Ada Hamosh for helpful discussions on the topic of locus homogeneity in disorders with autosomal-dominant inheritance. We thank Ms. Julia Fekecs for preparing the figures. We thank Drs. Alun Thomas and Doug Easton for helpful discussions.

Funding: L.G.B. was supported by NIH grant HG200328-17. S.V.T. was supported by NIH grant R01 CA264971. H.L.R. and S.M.H. were supported by NIH grant U24HG006834. This research was supported in part by the Intramural Research program of the NIH, National Human Genome Research Institute, and National Cancer Institute. The opinions and recommendations presented here represent those of the authors and the ClinGen consortium but do not necessarily reflect the views of the institutions to which we are affiliated.

Declaration of interests

L.G.B. is a member of the Illumina Medical Ethics Advisory Committee and receives research support from Merck, Inc. and honoraria from Cold Spring Harbor Laboratory Press and Wolters-Kluwer. H.L.R. receives funding from Illumina and Microsoft for rare disease research.

Received: April 14, 2023
Accepted: November 21, 2023
Published: December 15, 2023

References

1. Richards, S., Aziz, N., Bale, S., Bick, D., Das, S., Gastier-Foster, J., Grody, W.W., Hegde, M., Lyon, E., Spector, E., et al. (2015). Standards and guidelines for the interpretation of sequence variants: a joint consensus recommendation of the American College of Medical Genetics and Genomics and the Association for Molecular Pathology. Genet. Med. *17*, 405–424.
2. Tavtigian, S.V., Greenblatt, M.S., Harrison, S.M., Nussbaum, R.L., Prabhu, S.A., Boucher, K.M., Biesecker, L.G.; and ClinGen Sequence Variant Interpretation Working Group ClinGen SVI (2018). Modeling the ACMG/AMP variant classification guidelines as a Bayesian classification framework. Genet. Med. *20*, 1054–1060.
3. Rehm, H.L., Berg, J.S., Brooks, L.D., Bustamante, C.D., Evans, J.P., Landrum, M.J., Ledbetter, D.H., Maglott, D.R., Martin, C.L., Nussbaum, R.L., et al. (2015). ClinGen–the Clinical Genome Resource. N. Engl. J. Med. *372*, 2235–2242.
4. Tavtigian, S.V., Harrison, S.M., Boucher, K.M., and Biesecker, L.G. (2020). Fitting a naturally scaled point system to the ACMG/AMP variant classification guidelines. Hum. Mutat. *41*, 1734–1737.
5. Ioannidis, N.M., Rothstein, J.H., Pejaver, V., Middha, S., McDonnell, S.K., Baheti, S., Musolf, A., Li, Q., Holzinger, E., Karyadi, D., et al. (2016). REVEL: An Ensemble Method for Predicting the Pathogenicity of Rare Missense Variants. Am. J. Hum. Genet. *99*, 877–885.
6. Strande, N.T., Riggs, E.R., Buchanan, A.H., Ceyhan-Birsoy, O., DiStefano, M., Dwight, S.S., Goldstein, J., Ghosh, R., Seifert, B.A., Sneddon, T.P., et al. (2017). Evaluating the Clinical Validity of Gene-Disease Associations: An Evidence-Based Framework Developed by the Clinical Genome Resource. Am. J. Hum. Genet. *100*, 895–906.

7. Thompson, D., Easton, D.F., and Goldgar, D.E. (2003). A full-likelihood method for the evaluation of causality of sequence variants from family data. Am. J. Hum. Genet. *73*, 652–655.
8. Ott, J. (1999). Analysis of Human Genetic Linkage (Johns Hopkins University Press).
9. Gahl, W.A., Thoene, J.G., and Schneider, J.A. (2002). N. Engl. J. Med. *347*, 111–121.
10. Town, M., Jean, G., Cherqui, S., Attard, M., Forestier, L., Whitmore, S.A., Callen, D.F., Gribouval, O., Broyer, M., Bates, G.P., et al. (1998). A novel gene encoding an integral membrane protein is mutated in nephropathic cystinosis. Nat. Genet. *18*, 319–324.
11. Kalatzis, V., Cohen-Solal, L., Cordier, B., Frishberg, Y., Kemper, M., Nuutinen, E.M., Legrand, E., Cochat, P., and Antignac, C. (2002). Identification of 14 novel CTNS mutations and characterization of seven splice site mutations associated with cystinosis. Hum. Mutat. *20*, 439–446.
12. Rohlfs, E.M., Zhou, Z., Sugarman, E.A., Heim, R.A., Pace, R.G., Knowles, M.R., Silverman, L.M., and Allitto, B.A. (2002). The I148T CFTR allele occurs on multiple haplotypes: a complex allele is associated with cystic fibrosis. Genet. Med. *4*, 319–323.
13. Abou Tayoun, A.N., Pesaran, T., DiStefano, M.T., Oza, A., Rehm, H.L., Biesecker, L.G., Harrison, S.M.; and ClinGen Sequence Variant Interpretation Working Group ClinGen SVI (2018). Recommendations for Interpreting the Loss of Function PVS1 ACMG/AMP Variant Criterion. Hum. Mutat. *39*, 1517–1524.
14. Brnich, S.E., Abou Tayoun, A.N., Couch, F.J., Cutting, G.R., Greenblatt, M.S., Heinen, C.D., Kanavy, D.M., Luo, X., McNulty, S.M., Starita, L.M., et al. (2019). Recommendations for application of the functional evidence PS3/BS3 criterion using the ACMG/AMP sequence variant interpretation framework. Genome Med. *12*, 3.
15. Pejaver, V., Byrne, A.B., Feng, B.J., Pagel, K.A., Mooney, S.D., Karchin, R., O'Donnell-Luria, A., Harrison, S.M., Tavtigian, S.V., Greenblatt, M.S., et al. (2022). Calibration of computational tools for missense variant pathogenicity classification and ClinGen recommendations for PP3/BP4 criteria. Am. J. Hum. Genet. *109*, 2163–2177.
16. Laugel, V. (2013). Cockayne syndrome: the expanding clinical and mutational spectrum. Mech. Ageing Dev. *134*, 161–170.
17. Karikkineth, A.C., Scheibye-Knudsen, M., Fivenson, E., Croteau, D.L., and Bohr, V.A. (2017). Cockayne syndrome: Clinical features, model systems and pathways. Ageing Res. Rev. *33*, 3–17.
18. Calmels, N., Greff, G., Obringer, C., Kempf, N., Gasnier, C., Tarabeux, J., Miguet, M., Baujat, G., Bessis, D., Bretones, P., et al. (2016). Uncommon nucleotide excision repair phenotypes revealed by targeted high-throughput sequencing. Orphanet J. Rare Dis. *11*, 26.
19. Forsyth, R., and Gunay-Aygun, M. (1993). Bardet-Biedl Syndrome Overview. In GeneReviews((R)), M.P. Adam, D.B. Everman, G.M. Mirzaa, R.A. Pagon, S.E. Wallace, L.J.H. Bean, K.W. Gripp, and A. Amemiya, eds.
20. Shearer, A.E., Hildebrand, M.S., and Smith, R.J.H. (1993). Hereditary Hearing Loss and Deafness Overview. In GeneReviews((R)), M.P. Adam, D.B. Everman, G.M. Mirzaa, R.A. Pagon, S.E. Wallace, L.J.H. Bean, K.W. Gripp, and A. Amemiya, eds.
21. Baetens, M., Van Laer, L., De Leeneer, K., Hellemans, J., De Schrijver, J., Van De Voorde, H., Renard, M., Dietz, H., Lacro, R.V., Menten, B., et al. (2011). Applying massive parallel sequencing to molecular diagnosis of Marfan and Loeys-Dietz syndromes. Hum. Mutat. *32*, 1053–1062.
22. Northrup, H., Koenig, M.K., Pearson, D.A., and Au, K.S. (1993). Tuberous Sclerosis Complex. In GeneReviews((R)), M.P. Adam, D.B. Everman, G.M. Mirzaa, R.A. Pagon, S.E. Wallace, L.J.H. Bean, K.W. Gripp, and A. Amemiya, eds.
23. Oza, A.M., DiStefano, M.T., Hemphill, S.E., Cushman, B.J., Grant, A.R., Siegert, R.K., Shen, J., Chapin, A., Boczek, N.J., Schimmenti, L.A., et al. (2018). Expert specification of the ACMG/AMP variant interpretation guidelines for genetic hearing loss. Hum. Mutat. *39*, 1593–1613.
24. Bayrak-Toydemir, P., McDonald, J., Mao, R., Phansalkar, A., Gedge, F., Robles, J., Goldgar, D., and Lyon, E. (2008). Likelihood ratios to assess genetic evidence for clinical significance of uncertain variants: hereditary hemorrhagic telangiectasia as a model. Exp. Mol. Pathol. *85*, 45–49.
25. Jarvik, G.P., and Browning, B.L. (2016). Consideration of Cosegregation in the Pathogenicity Classification of Genomic Variants. Am. J. Hum. Genet. *98*, 1077–1081.
26. Mohammadi, L., Vreeswijk, M.P., Oldenburg, R., van den Ouweland, A., Oosterwijk, J.C., van der Hout, A.H., Hoogerbrugge, N., Ligtenberg, M., Ausems, M.G., van der Luijt, R.B., et al. (2009). A simple method for co-segregation analysis to evaluate the pathogenicity of unclassified variants; BRCA1 and BRCA2 as an example. BMC Cancer *9*, 211.
27. Biesecker, L.G., Adam, M.P., Alkuraya, F.S., Amemiya, A.R., Bamshad, M.J., Beck, A.E., Bennett, J.T., Bird, L.M., Carey, J.C., Chung, B., et al. (2021). A dyadic approach to the delineation of diagnostic entities in clinical genomics. Am. J. Hum. Genet. *108*, 8–15.
28. Campbell, P., Ellingford, J.M., Parry, N.R.A., Fletcher, T., Ramsden, S.C., Gale, T., Hall, G., Smith, K., Kasperaviciute, D., Thomas, E., et al. (2019). Clinical and genetic variability in children with partial albinism. Sci. Rep. *9*, 16576.
29. Ruiz-Perez, V.L., Tompson, S.W.J., Blair, H.J., Espinoza-Valdez, C., Lapunzina, P., Silva, E.O., Hamel, B., Gibbs, J.L., Young, I.D., Wright, M.J., and Goodship, J.A. (2003). Mutations in two nonhomologous genes in a head-to-head configuration cause Ellis-van Creveld syndrome. Am. J. Hum. Genet. *72*, 728–732.

The American Journal of Human Genetics, Volume *111*

Supplemental information

ClinGen guidance for use of the PP1/BS4 co-segregation and PP4 phenotype specificity criteria for sequence variant pathogenicity classification

Leslie G. Biesecker, Alicia B. Byrne, Steven M. Harrison, Tina Pesaran, Alejandro A. Schäffer, Brian H. Shirts, Sean V. Tavtigian, Heidi L. Rehm, and ClinGen Sequence Variant Interpretation Working Group

Table S1. Worksheet for apportioning evidence amongst two plausible candidate variants on an allele that is implicated by PP1 or PP4 evidence.

Var1 evidence criteria: ___________________________ (list criteria, e.g., PVS1_Moderate, but do NOT include PP1, PP4, or BS4 evidence

Var1 evidence points total: _________pts Line 1

Var1 posterior probability % (Table 2) based on points from Line 1 _________% Line 2

Var2 evidence criteria: ___________________________ (list criteria, e.g., PP3_Mod, but do NOT include PP1, PP4, or BS4 evidence

Var 2 evidence points total: _________pts Line 3

Var2 posterior probability % (Table 2) based on points from Line 1 _________% Line 4

Divide (Line 2)/(Line 4), (relative pathogenicity of Var1 to Var2) _________:1 Line 5

Enter the diagnostic yield of testing for this phenotype: _________% Line 6

Adjusted posterior for Var1 = (Line 6) * (Line 5)/((Line 5) +1) _________% Line 7

Convert Var1 adjusted posterior to points, using Table 2 _________pts Line 8

Adjusted posterior for Var2 = (Line 6) * (1/(Line 5)/(1/Line 5 +1) _________% Line 9

Convert Var2 adjusted posterior to points, using Table 2 _________pts Line 10

Error check: Add (Line 7) + (Line 9), this should be equal to Line 6 _________%
